# Interplay between interferon regulatory factor 1 and BRD4 in the regulation of PD-L1 in pancreatic stellate cells

**DOI:** 10.1038/s41598-018-31658-1

**Published:** 2018-09-05

**Authors:** Kazumi Ebine, Krishan Kumar, Thao N. Pham, Mario A. Shields, Katharine A. Collier, Meng Shang, Brian T. DeCant, Raul Urrutia, Rosa F. Hwang, Sam Grimaldo, Daniel R. Principe, Paul J. Grippo, David J. Bentrem, Hidayatullah G. Munshi

**Affiliations:** 1 Department of Medicine, Feinberg School of Medicine, Northwestern University, Chicago, IL USA; 2 Cold Spring Harbor Laboratory, Cold Spring Harbor, Cold Spring, NY USA; 3 Laboratory of Epigenetics and Chromatin Dynamics, Division of Gastroenterology and Hepatology, Department of Internal Medicine, Epigenomics Translational Program, Center for Individualized Medicine, Mayo Clinic, Rochester, MN USA; 4 Department of Surgical Oncology, The University of Texas MD Anderson Cancer Center, Houston, TX USA; 5 Department of Medicine, University of Illinois, Chicago, IL USA; 6 Department of Surgery, Feinberg School of Medicine, Northwestern University, Chicago, IL USA; 7 Jesse Brown VA Medical Center, Chicago, IL USA; 8 The Robert H. Lurie Comprehensive Cancer Center, Chicago, IL USA

## Abstract

The fibrotic reaction is a characteristic feature of human pancreatic ductal adenocarcinoma (PDAC) tumors. It is associated with activation and proliferation of pancreatic stellate cells (PSCs), which are key regulators of fibrosis *in vivo*. While there is increasing interest in the regulation of PD-L1 expression in cancer and immune cells, the expression and regulation of PD-L1 in other stromal cells, such as PSCs, has not been fully evaluated. Here we show that PSCs *in vitro* express higher PD-L1 mRNA and protein levels compared to the levels present in PDAC cells. We show that inhibitors targeting bromodomain and extra-terminal (BET) proteins and BRD4 knockdown decrease interferon-γ (IFN-γ)-induced PD-L1 expression in PSCs. We also show that c-MYC, one of the well-established targets of BET inhibitors, does not mediate IFN-γ-regulated PD-L1 expression in PSCs. Instead we show that interferon regulatory factor 1 (IRF1) mediates IFN-γ-induced PD-L1 expression in PSCs. Finally, while we show that BET inhibitors do not regulate IFN-γ-induced IRF1 expression in PSCs, BET inhibitors decrease binding of IRF1 and BRD4 to the PD-L1 promoter. Together, these results demonstrate the interplay between IRF1 and BRD4 in the regulation of PD-L1 in PSCs.

## Introduction

PD-L1 expression is seen in a number of human cancers, with increased expression of PD-L1 by cancer cells in some tumor types can be associated with responses to antibodies targeting PD-1/PD-L1^1^. For example, increased expression of PD-L1 in non-small cell lung cancers has predictive value for response to antibodies targeting PD-1/PD-L1^2,3^. However, the predictive value for response to immune checkpoint inhibitors in other tumor types, e.g., bladder cancer^4^, correlates with PD-L1 expression by infiltrating immune cells and not by cancer cells^1^. While there is increasing interest in identifying mechanisms regulating PD-L1 expression in cancer and immune cells, the expression and regulation of PD-L1 in other stromal cells, such as fibroblasts, has not been fully evaluated. Importantly, the stroma in some tumors can account for a significant portion of the tumor mass. For example, the stroma in human pancreatic ductal adenocarcinoma (PDAC) tumors, which can account for as much as 80–90% of the tumor mass, is associated with proliferation of pancreatic stellate cells (PSCs)^5,6^. The PSCs are the predominant fibroblasts present in human PDAC tumors and the key regulators of fibrosis *in vivo*^5^.

Recently, we showed that inhibitors targeting bromodomain (BRD) and extra-terminal (BET) proteins induce primary PSCs isolated from human PDAC tumors to become quiescent^7^. The BET proteins, which include BRD2, BRD3, BRD4, and the testis-specific BRDT, regulate transcription of genes involved in several human diseases^8,9^. These proteins bind to acetylation motifs present in histones and enable recruitment of transcription factors and other chromatin regulators during RNA transcription^8,9^. Importantly, a number of selective and potent small-molecule BET inhibitors are currently being evaluated in clinical trials for solid tumors^8,9^. In addition, inhibitors that promote degradation of BET proteins through proteolysis-targeting chimera (PROTAC) are also being developed^10–13^. While BET inhibitors were recently shown to repress PD-L1 expression in lymphoma and ovarian cancer cells^14,15^, the contribution of BET proteins to the regulation of PD-L1 expression in PSCs has yet to be evaluated.

In this report, we show that PSCs express higher PD-L1 mRNA and protein levels compared to the levels present in PDAC cells. We show that BET inhibitors, BET PROTAC and BRD4 knockdown decrease interferon-γ (IFN-γ)-induced PD-L1 expression in PSCs. We also show that c-MYC, one of the well-established targets of BET inhibitors^16–18^, does not mediate IFN-γ-mediated PD-L1 expression in PSCs. Instead we show that interferon regulatory factor 1 (IRF1) mediates IFN-γ-induced PD-L1 expression in PSCs. Finally, while we show that BET inhibitors do not regulate IFN-γ-induced IRF1 expression in PSCs, BET inhibitors decrease binding of IRF1 and BRD4 to the PD-L1 promoter. Together, these results demonstrate the interplay between IRF1 and BRD4 in the regulation of PD-L1 in PSCs.

## Results

### PSCs express PD-L1

**Figure 1:**
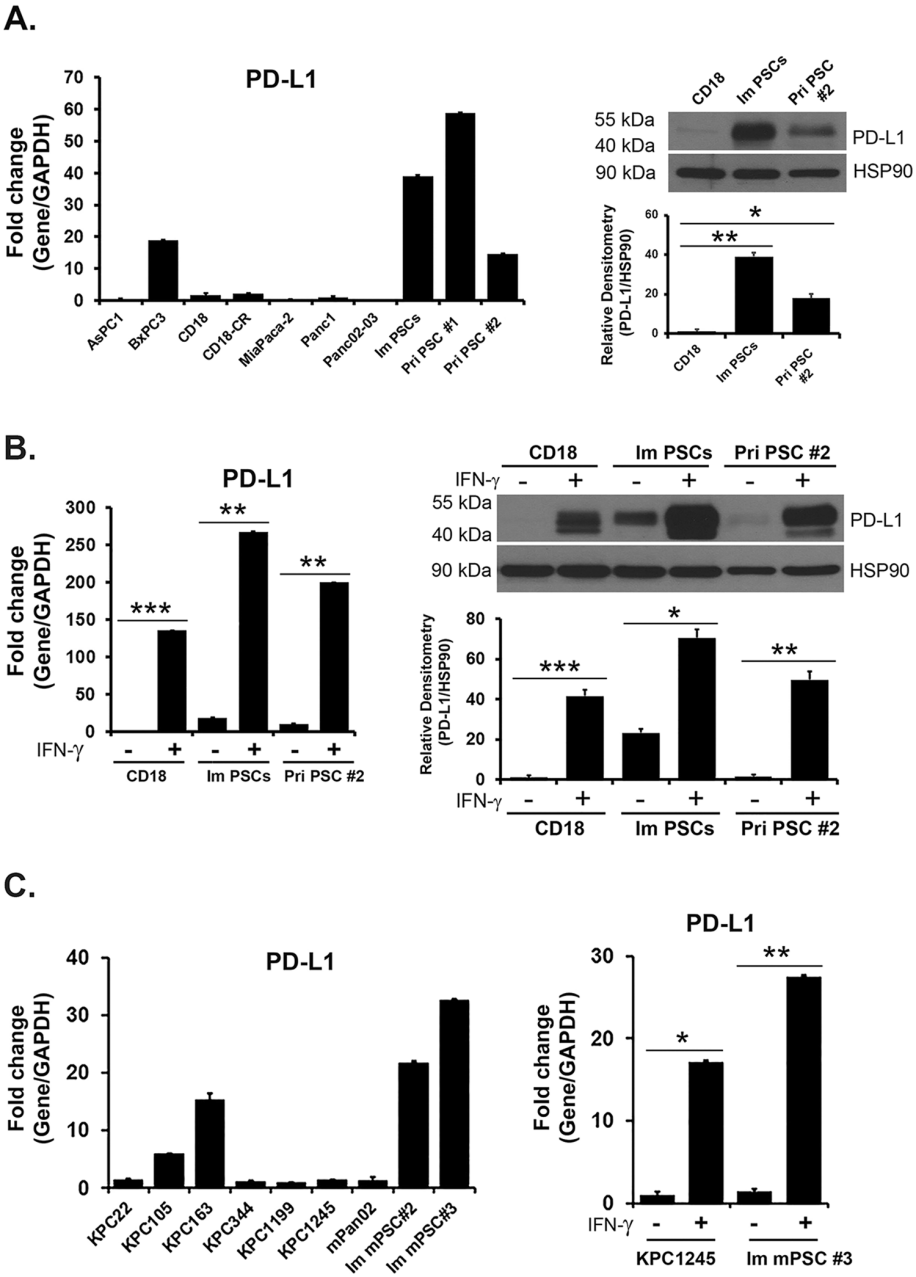
Pancreatic stellate cells (PSCs) express PD-L1. (**A**) Human *PD-L1* mRNA expression was analyzed in a panel of human PDAC cell lines, in an immortalized human pancreatic stellate cell line (Im PSCs), and in primary pancreatic stellate cells (Pri PSCs) isolated from human PDAC tumors. The relative expression was normalized to *PD-L1* mRNA levels present in the Panc1 cells. PD-L1 protein expression was determined in CD18 PDAC cells, in Im PSCs and in Pri PSC #2 using HSP90 as loading control. PD-L1 and HSP90 bands from three independent experiments were quantified by densitometry and data expressed as relative protein ratios. (**B**) CD18 PDAC cells, Im PSCs, and Pri PSC #2 were treated with IFN-γ (0.2 μg/mL) for 24 hours. The effect on *PD-L1* mRNA expression was determined by qRT-PCR and the effect on PD-L1 protein expression was determined by Western blotting. PD-L1 and HSP90 bands from three independent experiments were quantified by densitometry and data expressed as relative protein ratios. (**C**) Mouse *PD-L1* mRNA was analyzed in a panel of mouse PDAC cell lines established from tumors arising in the KPC (Kras/p53) mouse model, in mouse Pan02 cells and in two immortalized mouse pancreatic stellate cell lines (Im mPSCs). The relative expression was normalized to mouse *PD-L1* mRNA levels present in the KPC1199 cells. KPC1245 PDAC cells and Im mPSC #3 were treated with mouse IFN-γ (0.05 μg/mL) for 4 hours. The effect on mouse *PD-L1* mRNA expression was determined by qRT-PCR. The gene expression results are representative of three independent experiments. Bar graphs represent means +/−S.D. *p < 0.05; **p < 0.01; ***p < 0.001 relative to control samples.

Since activated PSCs are the predominant fibroblasts present in the pancreatic stromal reaction^5^, and the immune cells are very likely to encounter these cells *in vivo*, we evaluated expression of PD-L1 in an immortalized PSC cell line and in primary PSCs isolated from human PDAC tumors using the outgrowth assay^19^. Human PSCs express *PD-L1* mRNA, which in most cases exceeded the *PD-L1* mRNA levels present in human PDAC cell lines (Fig. 1A). In agreement with the mRNA findings, both the immortalized PSC cell line and the primary PSCs demonstrated higher PD-L1 protein levels relative to the levels present in the CD18 PDAC cell line (Fig. 1A). However, treatment with IFN-γ resulted in robust induction of PD-L1 in CD18 cells to levels comparable to basal PD-L1 levels present in PSCs (Fig. 1B).

We also evaluated the expression of *PD-L1* mRNA expression in a panel of mouse PDAC cell lines and in 2 immortalized mouse PSC cell lines. Similar to our findings with human PDAC and PSC cells (Fig. 1A), mouse PSC cells show relatively increased *PD-L1* mRNA levels compared to levels present in mouse Pan02 PDAC cell line and in mouse PDAC cell lines generated from tumors arising in the KPC transgenic mouse model of pancreatic cancer (Fig. 1C). Similarly, treatment of mouse PDAC cells with mouse IFN-γ resulted in induction of mouse *PD-L1* mRNA to levels comparable to *PD-L1* mRNA levels present in mouse PSC cell lines (Fig. 1C).

### BET inhibitors decrease PD-L1 expression in PDAC cells and PSCs

**Figure 2:**
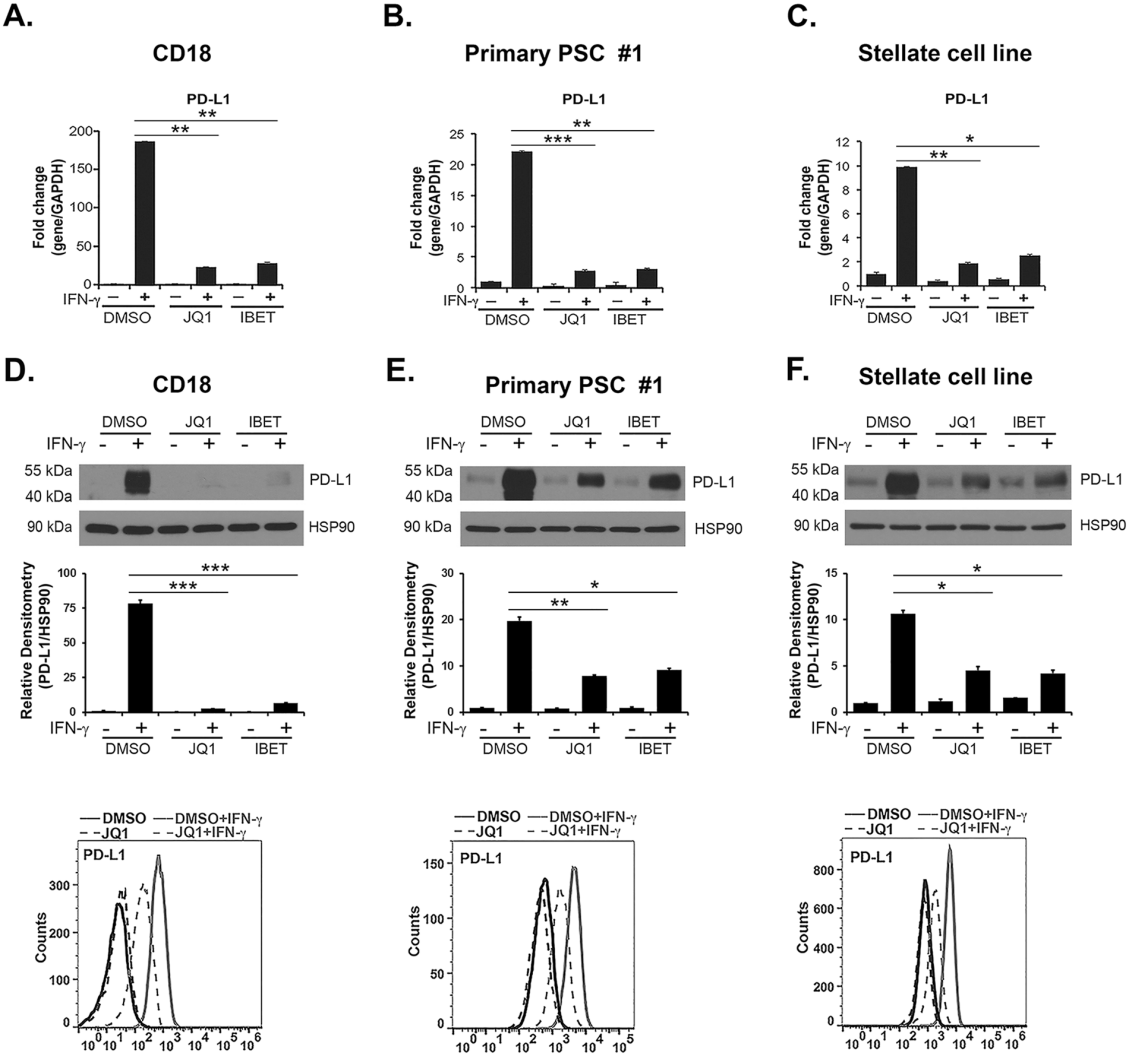
BET inhibitors decrease IFN-γ-induced PD-L1 expression in PDAC cells and PSCs. CD18 PDAC cells, primary PSC #1, and the pancreatic stellate cell line were pre-treated with the BET inhibitors JQ1 (1 μM) or I-BET151 (I-BET; 1 μM) for 30 minutes and treated with IFN-γ (0.2 μg/mL) for 24 hours. (**A**–**C**) The effect on *PD-L1* mRNA expression was determined by qRT-PCR. (**D**–**F**) The effect on PD-L1 protein expression was determined by Western blotting and by FACS analysis. PD-L1 and HSP90 bands from three independent experiments were quantified by densitometry and data expressed as relative protein ratios. The gene expression results are representative of five independent experiments. Bar graphs represent means +/−S.D. *p < 0.05; **p < 0.01; ***p < 0.001 relative to control samples.

Recently, we showed that BET inhibitors can modulate stellate cell function by inducing primary PSCs to become quiescent, with decreased α-SMA and collagen I expression^7^. Thus, we evaluated the effect of BET inhibitors JQ1 and I-BET151 on PD-L1 expression in PSCs and in PDAC cells. JQ1 and I-BET151 decreased IFN-γ-induced PD-L1 expression at the mRNA and protein levels in CD18 and AsPC1 PDAC cells (Fig. 2A and D, and Supplemental Fig. 1A). BET inhibitors also decreased IFN-γ-induced PD-L1 mRNA and protein expression in primary PSCs isolated from human PDAC tumors (Fig. 2B and E, and Supplemental Fig. 1B) and in the stellate cell line (Fig. 2C and F). Significantly, IFN-γ rapidly induced PD-L1 expression in PSCs and in PDAC cells, which was inhibited by pre-treatment with JQ1 (Supplemental Fig. 1C).

### BET PROTAC decreases PD-L1 expression in PSCs

**Figure 3:**
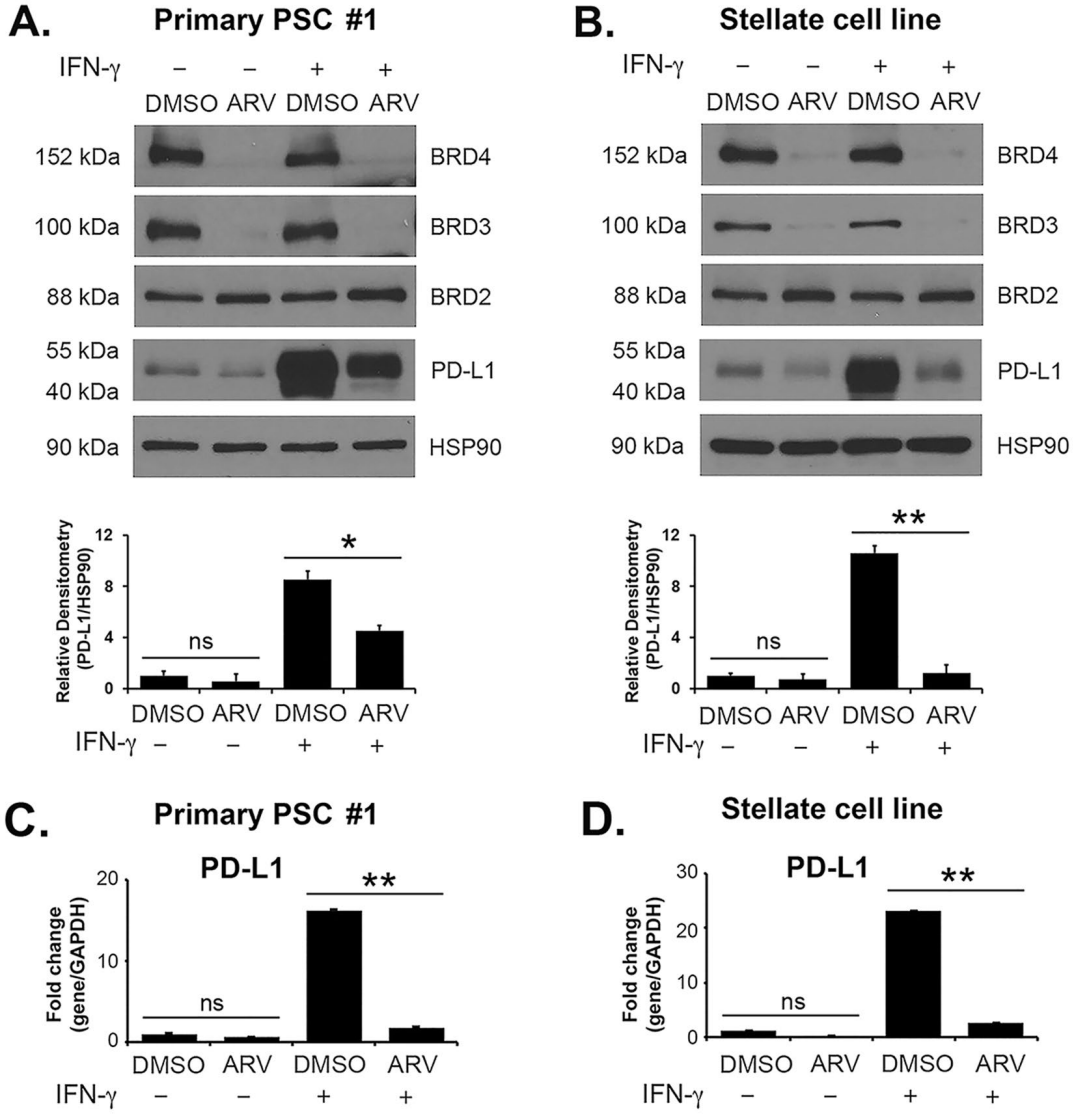
BET PROTAC ARV-825 decreases IFN-γ-induced PD-L1 expression in PSCs. Primary PSC #1 and the pancreatic stellate cell line were pre-treated with the BET PROTAC ARV-825 (0.25 μM) for 30 minutes and treated with IFN-γ (0.2 μg/mL) for 24 hours. (**A**,**B**) The effect on BRD2, BRD3, BRD4 and PD-L1 protein expression was determined by Western blotting. PD-L1 and HSP90 protein bands from three independent experiments were quantified by densitometry and data expressed as relative protein ratios. (**C**,**D**) The effect on *PD-L1* mRNA expression was determined by qRT-PCR. The gene expression results are representative of three independent experiments. Bar graphs represent means +/−S.D. ns, not significant; *p < 0.05; **p < 0.01 relative to control samples.

We also evaluated the effect of BET PROTAC, which can promote degradation of BET proteins^10–13^, on PD-L1 expression in PSCs. Treatment with the BET PROTAC ARV-825 resulted in loss of BRD3 and BRD4 proteins without decreasing BRD2 protein levels. Significantly, ARV-825 decreased IFN-γ-induced PD-L1 mRNA and protein expression in primary PSCs and in the stellate cell line (Fig. 3A–D).

### BRD4 regulates *PD-L1* expression in PSCs

**Figure 4:**
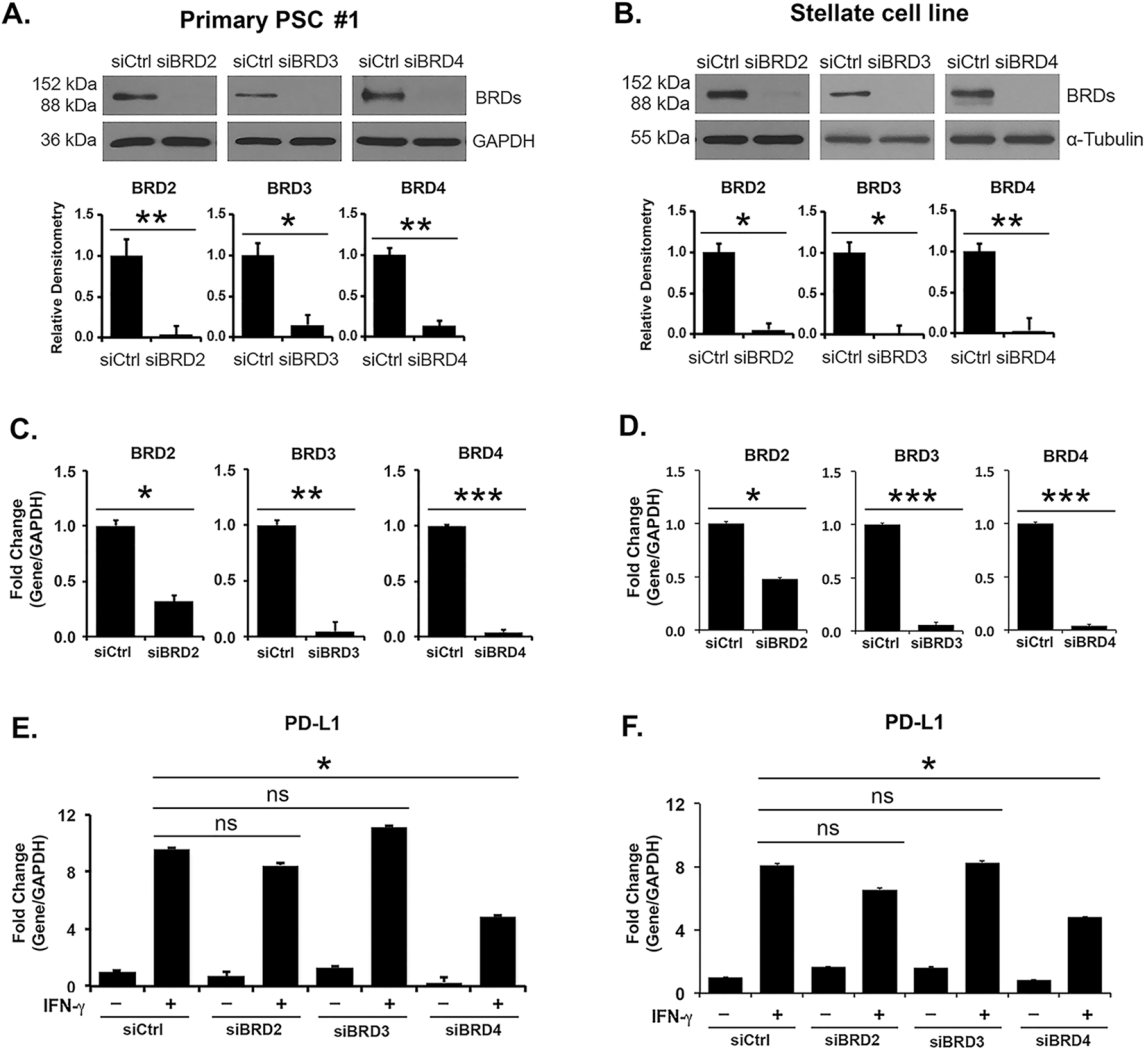
BRD4 knockdown decreases IFN-γ-induced *PD-L1* expression in PSCs. (**A**,**B**) Primary PSC #1 and the pancreatic stellate cell line were transfected with control siRNA or with siRNAs against BRD2, BRD3 or BRD4 for 48 hours. The cells were then treated with IFN-γ (0.2 μg/mL) for 24 hours. (**A**,**B**) The effect on the individual BRD proteins was determined by Western blotting. Bands from three independent experiments were quantified by densitometry and data expressed as relative ratios. (**C**,**D**) The effect on the individual *BRD* mRNAs was determined by qRT-PCR. (**E**,**F**) The effect on *PD-L1* mRNAs was determined by qRT-PCR. The gene expression results are representative of four independent experiments. Bar graphs represent means +/− S.D. ns, not significant; *p < 0.05; **p < 0.01; ***p < 0.001 relative to control samples.

We next evaluated the relative contribution of the BET proteins BRD2, BRD3 and BRD4 to the regulation of IFN-γ-induced PD-L1 expression in primary PSCs and in the stellate cell line. Knocking down BRD2 or BRD3 had minimal effects on IFN-γ-induced *PD-L1* mRNA in both primary PSCs (Fig. 4A,C and E, and Supplemental Fig. 2) and the stellate cell line (Fig. 4B,D and F). In contrast, BRD4 knockdown decreased IFN-γ-induced *PD-L1* mRNA expression in primary PSCs and in the stellate cell line (Fig. 4 and Supplemental Fig. 2).

### c-MYC knockdown does not suppress IFN-γ-induced *PD-L1* expression in PSCs and PDAC cells

**Figure 5:**
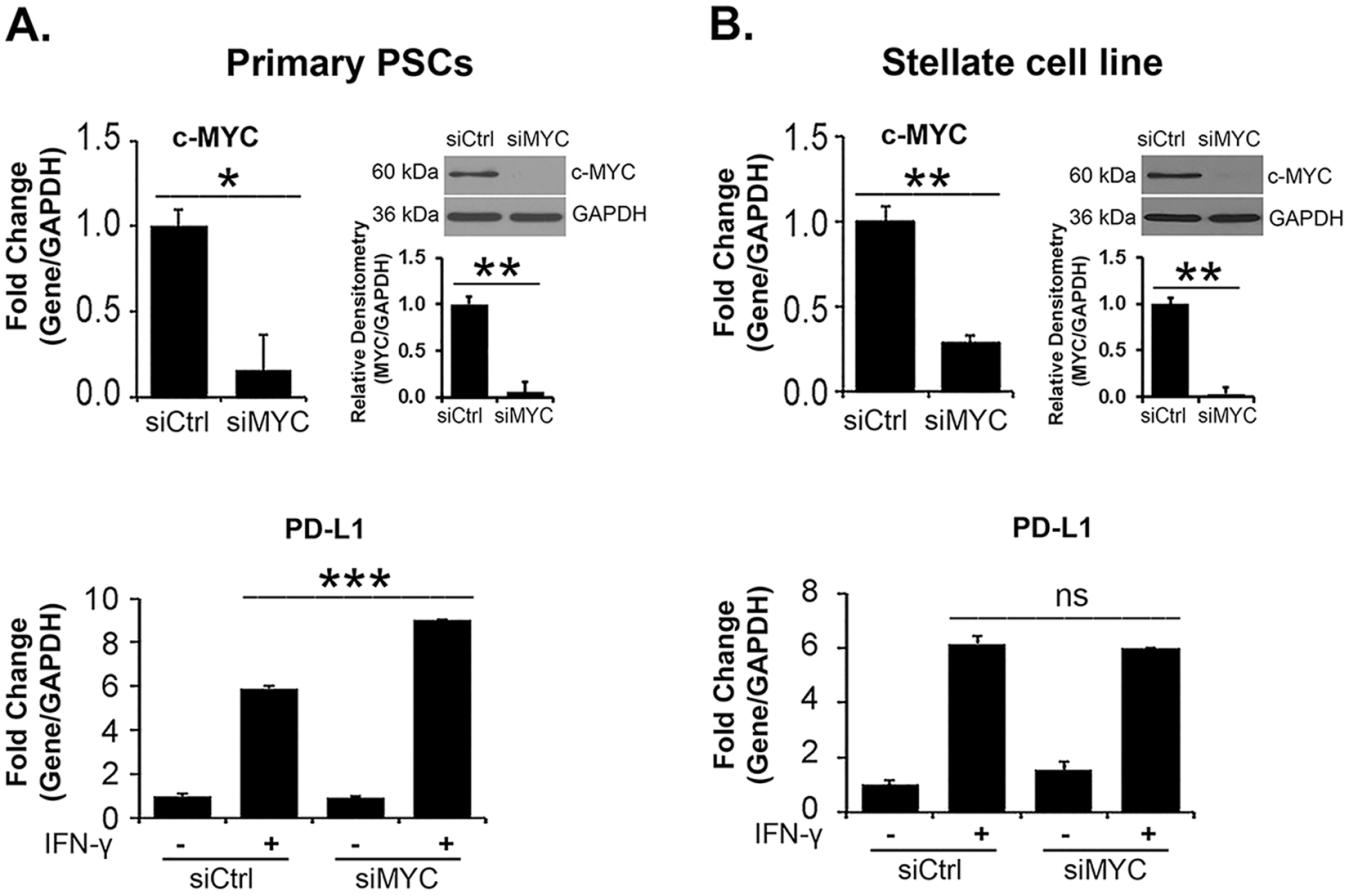
c-MYC knockdown does not decrease IFN-γ-induced *PD-L1* expression. (**A**,**B**) Primary PSCs and the pancreatic stellate cell line were transfected with control siRNA or with siRNA against c-MYC for 48 hours. The cells were then treated with IFN-γ (0.2 μg/mL) for 24 hours. The effect on c-MYC was determined by qRT-PCR and by Western blotting, and the effect on *PD-L1* mRNA was determined by qRT-PCR. c-MYC and GAPDH bands from three independent experiments were quantified by densitometry and data expressed as relative ratios. The gene expression results are representative of three independent experiments. Bar graphs represent means +/− S.D. ns, not significant; *p < 0.05; **p < 0.01; ***p < 0.001 relative to control samples.

Recently, c-MYC was shown to regulate PD-L1 expression in mouse and human tumor cells^20,21^, with suppression of c-MYC decreasing PD-L1 mRNA and protein levels. Since we have published that BET inhibitors can repress c-MYC levels both in the stellate cell line and in PDAC cells^7,22,23^, we evaluated the role of c-MYC in IFN-γ-induced *PD-L1* mRNA expression. Suppressing c-MYC in primary PSCs and in the stellate cell line did not decrease IFN-γ-induced *PD-L1* expression (Fig. 5A and B). Similarly, c-MYC knockdown in CD18 and AsPC1 PDAC cells did not decrease IFN-γ-induced *PD-L1* expression (Supplemental Fig. 3A). Significantly, c-MYC knockdown in fact increased *PD-L1* expression in some of these cells (Fig. 5A and Supplemental Fig. 3A). These results suggest that c-MYC does not mediate the suppressive effects of BET inhibitors on IFN-γ-induced *PD-L1* in PSCs and PDAC cells.

### IRF1 knockdown decreases IFN-γ-induced *PD-L1* expression

**Figure 6:**
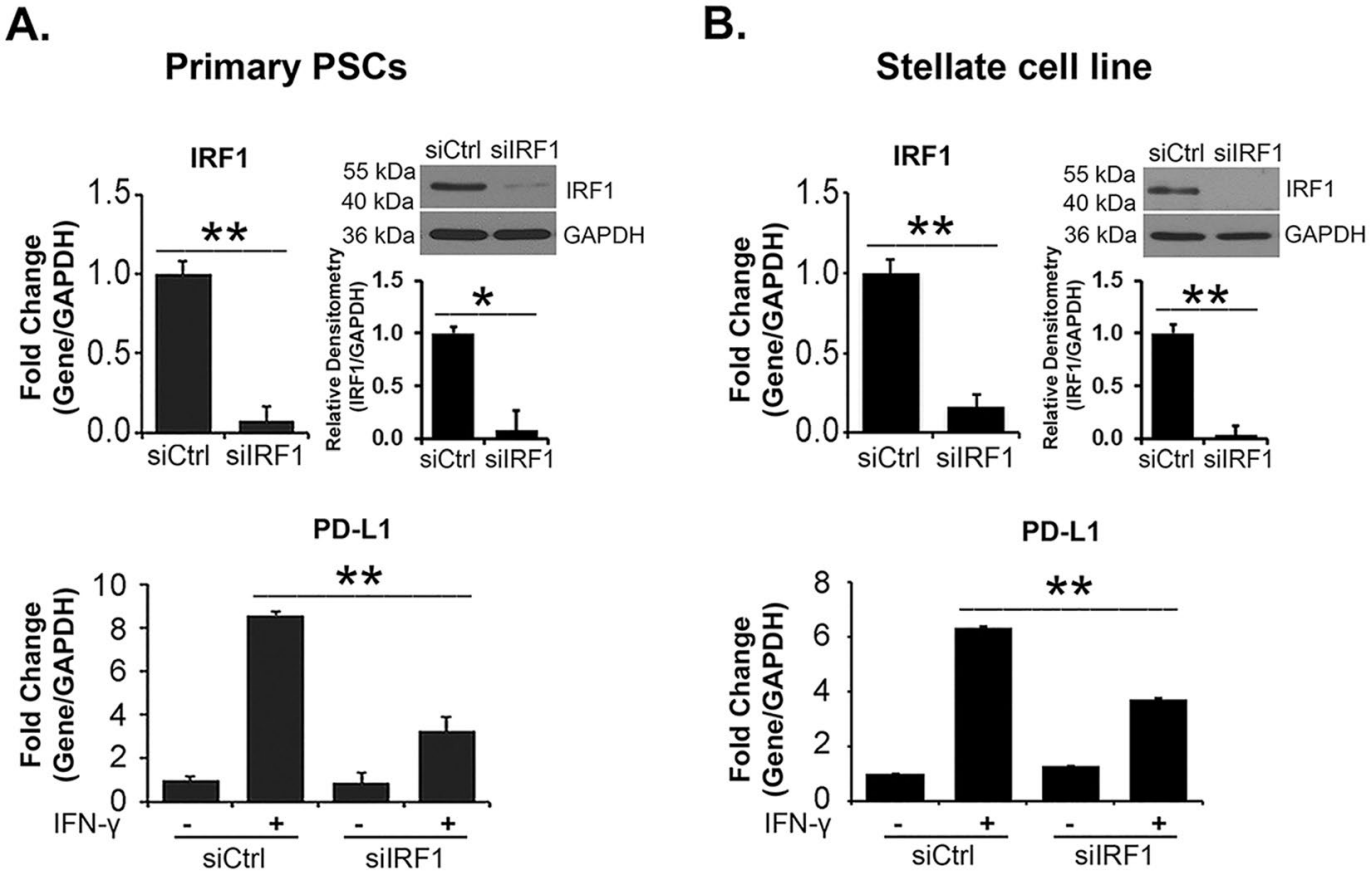
IRF1 knockdown decreases IFN-γ-induced *PD-L1* expression. (**A**,**B**) Primary PSCs and the pancreatic stellate cell line were transfected with control siRNA or with siRNA against IRF1 for 48 hours. The cells were then treated with IFN-γ (0.2 μg/mL) for 24 hours. The effect on IRF1 was determined by qRT-PCR and by Western blotting, and the effect on *PD-L1* mRNA was determined by qRT-PCR. IRF1 and GAPDH bands from three independent experiments were quantified by densitometry and data expressed as relative ratios. The gene expression results are representative of three independent experiments. Bar graphs represent means +/−S.D. ns, not significant; *p < 0.05; **p < 0.01 relative to control samples.

Previously, it was shown that interferon regulatory factor 1 (IRF1) regulated IFN-γ-induced PD-L1 expression in lung, liver and colon cancer cells^24^. Thus, we evaluated the extent to which BET inhibitors regulated IRF1 in PSCs and PDAC cells. Initially, we evaluated the role of IRF1 on IFN-γ-induced PD-L1 expression in PSCs. Knockdown of IRF1 decreased IFN-γ-induced PD-L1 expression in primary PSCs (Fig. 6A) and in the stellate cell line (Fig. 6B).

### BET inhibitors do not suppress IFN-γ-induced IRF1 protein expression

**Figure 7:**
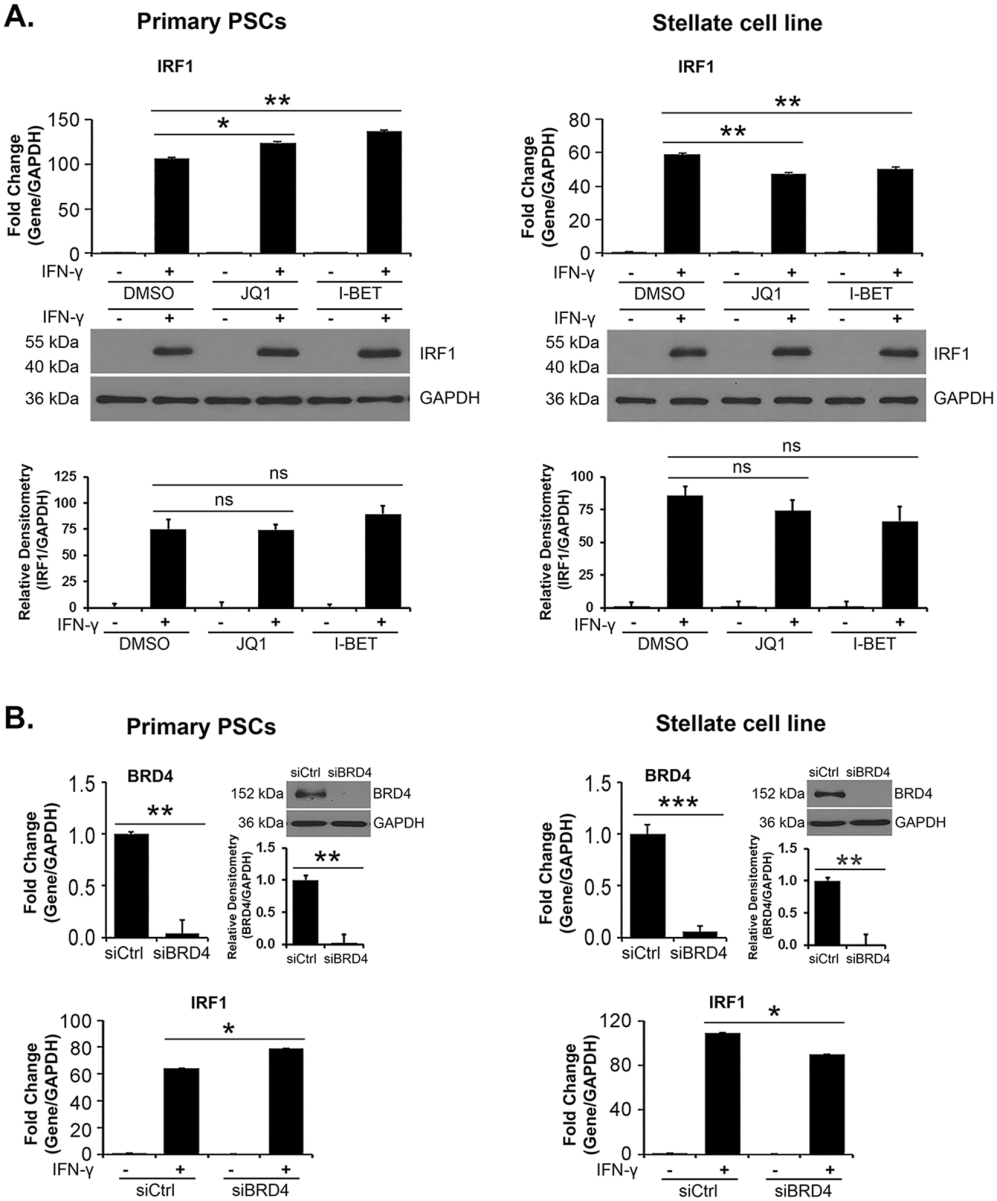
BET inhibitors do not suppress IFN-γ-induced IRF1 protein expression. (**A**) Primary PSCs and the pancreatic stellate cell line were pre-treated with the BET inhibitors JQ1 (1 μM) or I-BET151 (I-BET; 1 μM) for 30 minutes and then treated with IFN-γ (0.2 μg/mL) for 4 hours. The effect on IRF1 mRNA and protein expression was determined by qRT-PCR and by Western blotting. (**B**) Primary PSCs and the pancreatic stellate cell line were transfected with control siRNA or with siRNA against BRD4 for 48 hours. The cells were then treated with IFN-γ (0.2 μg/mL) for 4 hours. The effect on BRD4 protein was determined by qRT-PCR and by Western blotting, and the effect on *IRF1* mRNA was determined by qRT-PCR. Bands from three independent experiments were quantified by densitometry and data expressed as relative ratios. The gene expression results are representative of three independent experiments. Bar graphs represent means +/− S.D. ns, not significant; *p < 0.05; **p < 0.01; ***p < 0.001 relative to control samples.

We next evaluated the effects of JQ1 and I-BET151 on IFN-γ-induced IRF1 expression. While IFN-γ increased IRF1 expression, pre-treatment with JQ1 or I-BET151 had minimal effects on IFN-γ-induced IRF1 expression in primary PSCs and in the stellate cell line (Fig. 7A). BET inhibitors also had minimal effects on IFN-γ-induced IRF1 expression in the PDAC cell lines CD18 and AsPC1 (Supplemental Fig. 3B). Moreover, pre-treatment with the BET PROTAC ARV-825 (Supplemental Fig. 3C), or knockdown of BRD4 (Fig. 7B), also had minimal effects on IFN-γ-induced *IRF1* expression in primary PSCs and the stellate cell line.

### BET inhibitors decrease binding of IRF1 to *PD-L1* promoter

**Figure 8:**
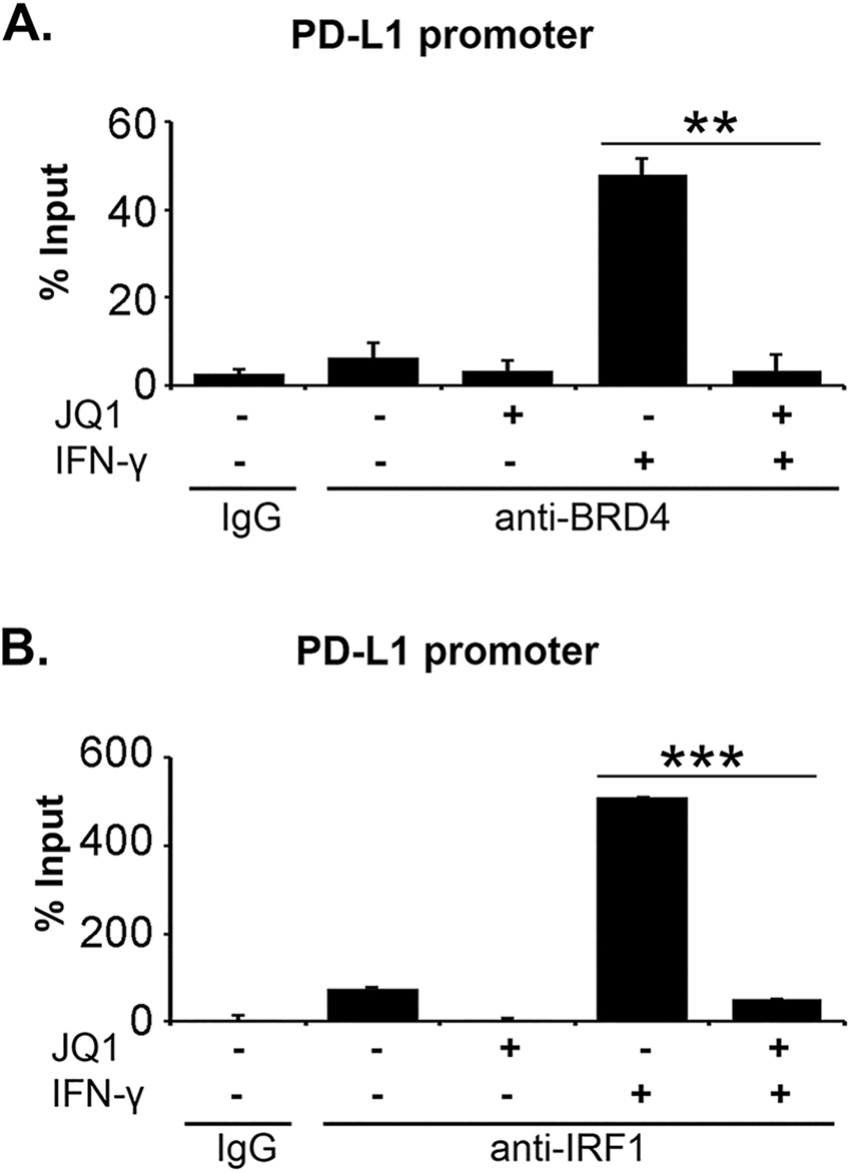
BET inhibitors decrease binding of IRF1 to PD-L1 promoter. (**A**, **B**) The pancreatic stellate cell line was pre-treated with the BET inhibitor JQ1 (1 μM) for 30 minutes and then treated with IFN-γ (0.2 μg/mL) for 4 hours. The cells were subjected to ChIP analysis using anti-BRD4 antibody (*A*) or anti-IRF1 antibody (*B*). An isotype-matched IgG was used as a negative control. The association with the *PD-L1* gene promoter was quantified by qPCR. The results are representative of three independent experiments. Bar graphs represent means +/−S.D. **p < 0.01; ***p < 0.001 relative to control samples.

Since BRD4 can bind to the PD-L1 promoter in cancer cells^14,15^, we initially evaluated the effect of BET inhibitors on the recruitment of BRD4 to the PD-L1 promoter in the stellate cell line. Treatment with IFN-γ increased binding of BRD4 to the PD-L1 promoter in the stellate cell line, which was inhibited in the presence of JQ1 (Fig. 8A). We next evaluated the effect of BET inhibitors on binding of IRF1 to the PD-L1 promoter in PSCs. As previously shown^15^, treatment with IFN-γ increased binding of IRF-1 to the PD-L1 promoter (Fig. 8B). Significantly, while JQ1 did not decrease IFN-γ-induced IRF1 protein expression (Fig. 7A), JQ1 decreased IRF1 binding to the PD-L1 promoter (Fig. 8B).

## Discussion

Since PD-L1 levels can correlate with response to immune checkpoint therapy in certain tumors^2,3^, there is increasing interest in understanding PD-L1 regulation^1^. However, while PD-L1 regulation has been extensively studied in cancer cells^1^, the regulation of PD-L1 in stromal cells is less well understood. Also, since the stroma in PDAC tumors predominates^5,6^, it is very likely that cytolytic T cells in PDAC tumors will encounter PSCs. In this report we show that PSCs, which contribute to the pronounced stroma that is seen in human PDAC tumors^5,6^, express PD-L1 and that BET inhibitors can repress PD-L1 in PSCs. Our data are in agreement with recent studies demonstrating that BET inhibitors can decrease PD-L1 expression in lymphoma and ovarian cancer cells^14,15^. In this report we also show that BET PROTAC and knockdown of BRD4, but not knockdown of BRD2 or BRD3, decrease PD-L1 expression in PSCs.

We have previously shown that BET inhibitors can target c-MYC in a human stellate cell line and in PDAC cells^7,22,23^. However, we have found that targeting c-MYC does not decrease PD-L1 levels in PSCs or in PDAC cell lines, indicating that the effect of BET inhibitors in these cells is not indirect through c-MYC repression. In fact, c-MYC knockdown in PDAC cells increased IFN-γ-induced PD-L1 expression, in agreement with a recent study showing that c-MYC knockdown in hepatocellular carcinoma cells results in increased IFN-γ-induced PD-L1 expression^25^. While our findings contrast with a study demonstrating that targeting c-MYC by shRNA or JQ1 treatment decreases PD-L1 in melanoma and lung cancer cells^20,21^, our findings are in agreement with another study showing that JQ1 regulation of PD-L1 in lymphoma cells is not mediated by c-MYC^15^. Instead, BET inhibitors directly decrease binding of BRD4 to the PD-L1 promoter in lymphoma cells^15^. We have also found that BET inhibitors directly decrease binding of BRD4 to the PD-L1 promoter in PSCs.

We also demonstrate that BET inhibitors decrease binding of IRF1 to the PD-L1 promoter in PSCs. IRF1 is well known to regulate PD-L1 in a number of different cancer types^24,26^. IRF1 rapidly binds to the PD-L1 promoter following IFN-γ treatment and down-regulation of IRF1 decreases PD-L1 expression^24^. We also show that IRF1 regulates PD-L1 expression in PSCs. Significantly, we have found that BET inhibitors, BET PROTAC and BRD4 siRNA do not decrease IRF1 protein expression in PSCs. Instead, we show that BET inhibitors decrease binding of IRF1 protein to the PD-L1 promoter. Our results are in contrast to the data in lymphoma cells in which BET inhibitors did not affect binding of IRF1 to the PD-L1 promoter^15^. It is possible that the effect of BET inhibitors on IRF1 binding to the PD-L1 promoter may vary depending on the cell type.

While the role of fibrosis in PDAC progression is controversial, and whether the pancreatic stroma is pro- or anti-tumorigenic continues to be debated^27–29^, recent reports have shown that normalizing the stroma^30^, instead of eliminating the stroma^28,29^, can enhance drug delivery and improve survival. We have recently also shown that BET inhibitors can normalize the stroma by inducing stellate cells to become quiescent as reflected by re-accumulation of lipid droplets, decreased collagen production and decreased α-SMA expression^7^. As we now show that BET inhibitors decrease PD-L1 expression, and since BET inhibitors can enhance response to anti-PD-1 antibody in MYC-expressing lymphoma tumors^15^, it is possible that BET inhibitors may also enhance response to immune checkpoint inhibitors in pancreatic cancer. However, since BET inhibitors decrease PD-L1, and thereby decrease the ‘target’ for the PD-1/PD-L1 checkpoint inhibitors, in future studies we will evaluate the relative efficacy of combining BET inhibitors with anti-PD-1/PD-L1 antibodies or with other immune checkpoint inhibitors, such as anti-CTLA4 antibodies^31^, in transgenic mouse models of pancreatic cancer.

In summary, we show that PSCs, the predominant fibroblasts in human PDAC tumors^5,6^, express high levels of PD-L1 and that the interplay between IRF1 and BRD4 regulates PD-L1 expression in PSCs. These results, together with our earlier studies evaluating the effect of BET inhibitors on pancreatic cancer cells and PSCs^7,22^, provide impetus for the evaluation of BET inhibitors in patients with pancreatic cancer.

## Methods

### Chemicals/reagents

General tissue culture materials were obtained from VWR International. Antibodies against BRD2 (ab139690), BRD3 (ab50818), BRD4 (ab128874) and IRF1 (ab26109) antibody for chromatin immunoprecipitation (ChIP) were obtained from Abcam. Antibodies against c-MYC (5605), human PD-L1 (E1L3N clone; 13684), IRF1 (8478) and control IgG (2729) antibody for ChIP were purchased from Cell Signaling Technology. Anti-BRD4 (A301–985A) antibody for ChIP was obtained from Bethyl Laboratories. PE-conjugated human PD-L1 (MIH1 clone; 12-5983-42) antibody was from Thermo Fisher. The GAPDH (MAB374) antibody was from Millipore, and α-tubulin (sc-8035) antibody and HSP90 (sc-7940) antibody were obtained from Santa Cruz Biotechnology. Secondary anti-mouse IgG (A4416) and anti-rabbit IgG (A6667) antibodies were purchased from Sigma. The BET inhibitors JQ1 and I-BET151 were obtained from Tocris Bioscience, the BET PROTAC ARV-825 was obtained from MedChem Express, and human and mouse IFN-γ was purchased from Thermo Fisher Scientific. The siRNAs against BRD2, BRD3, BRD4, c-MYC, and IRF1 were purchased from Thermo Fisher Scientific.

### Cell culture

Human PDAC cell lines AsPC1, BxPC3, CD18/HPAF-II, MiaPaca2, Panc1 and Panc02–03 were obtained from ATCC. Chemo-resistant CD18 (CD18-CR) cells were generated by treating parental CD18 cells with increasing concentration of 5-fluorouracil (5-FU) over a period of 3 months^32^. KPC22, KPC105, KPC163 and KPC344 – all derived from PDAC tumor developing in the KPC (Kras/p53) mouse model in B6 background – were obtained from Sam Grimaldo (University of Illinois, Chicago, US). KPC1245 and KPC1199 cells, both derived from KPC tumors developing in B6 background, were provided by David Tuveson (Cold Spring Harbor Laboratories, Cold Spring Harbor, New York, US)^33,34^. Mouse Pan02 cells were purchased from the NCI.

A human pancreatic stellate cell line, which was immortalized with telomerase and SV40 large T antigen^35–37^, was obtained from Dr. Rosa F. Hwang (MD Anderson Cancer Center, Houston, Texas, US). The immortalized mouse pancreatic stellate cell lines mPSC#2 and mPSC#3, which were immortalized with SV40 large T antigen^38^, were obtained from Dr. Raul Urrutia (Mayo, Rochester, Minnesota, US). Primary PSCs were generated from de-identified fresh human PDAC specimens using primary outgrowth cultures, which have been shown to be a reliable source of human pancreatic stellate cells^19^. The primary PSCs were used within 5–6 passages from isolation.

### Transfection

The human stellate cell line and the primary PSCs were transfected with siRNA against BRD2, BRD3, BRD4, c-MYC, IRF1 or control siRNA (20 nM) using RNAimax (Thermo Fisher Scientific) according to the manufacturer’s instructions^23,32^.

### qRT-PCR analysis

Quantitative gene expression was performed with gene-specific TaqMan probes, TaqMan Universal PCR Master Mix, and the 7500 Fast Real-time PCR System from Applied Biosystems. The data were then quantified with the comparative *C*_T_ method for relative gene expression^23,32^.

### ChIP

The human stellate cell line was pre-treated with JQ1 for 30 minutes, treated with IFN-γ for 4 hours, and then treated with formaldehyde to create DNA-protein cross-links. Chromatin fragments were prepared using EZ-Zyme Chromatin Prep kit (17–375, Millipore) and ChIP performed using the EZ-Magna ChIP A/G Chromatin Immunoprecipitation kit (17–10086, Millipore) and anti-BRD4 antibody (A301-985A, Bethyl Laboratories), anti-IRF1 antibody (ab26109, Abcam) or control IgG antibody (2729, Cell Signaling). Purified DNA was then analyzed by PCR using KiCqStart SYBR Green qPCR ReadyMix (KCQS02, Sigma) and primers specific for the PD-L1 locus: forward 5′-AAGCCATATGGGTCTGCTC-3′ and reverse 5′-TTATCAGAAAGGCGTCCCCC-3′^15^.

### Immunoblotting

Immunoblotting for BRD2, BRD3, BRD4, c-MYC, IRF1, PD-L1, α-tubulin, GAPDH and HSP90 was done as described previously^23,32^.

### Flow Cytometric Analysis

PDAC cells and PSCs in suspension were incubated with PE-conjugated anti-PD-L1 antibody and co-stained with DAPI prior to analysis with BD LSR Fortessa 6-Laser flow cytometer^7,32^.

### Study Approval

Pancreatic tissue was obtained from patients with PDAC undergoing resection on a protocol approved by the Institutional Review Board of Northwestern University. Informed consent was obtained from patients prior to resection. The resected specimens were deidentified and subsequently processed for isolation of primary PSCs. All experiments were performed in accordance with relevant guidelines and regulations.

### Statistical analysis

All statistical analyses were done using Microsoft Excel using a two-tailed *t*-test analysis. Error bars represent standard deviation.

## Electronic supplementary material

Supplemental Figures 1-3

## Data Availability

All data supporting the findings of this study are available with the article or from corresponding authors upon reasonable request.
