# Supplementary material for: Interplay between interferon regulatory factor 1 and BRD4 in the regulation of PD-L1 in pancreatic stellate cells
Source: Sci Rep. 2018 Sep 5;8:13225. doi: 10.1038/s41598-018-31658-1 (PMC6125340; doi:10.1038/s41598-018-31658-1)

Interplay between interferon regulatory factor 1 and BRD4 in the regulation of PD-L1 in pancreatic stellate cells

Kazumi Ebine[1,7,9], Krishan Kumar[1,8,9,*], Thao N. Pham[1], Mario A. Shields[2], Katharine A. Collier[1], Meng Shang[1,7], Brian T. DeCant[1], Raul Urrutia[3], Rosa F. Hwang[4], Sam Grimaldo[5], Daniel R. Principe[5], Paul J. Grippo[5], David J. Bentrem[6,7,8], and Hidayatullah G. Munshi[1,7,8,*]

[1]Department of Medicine, Feinberg School of Medicine, Northwestern University, Chicago, IL, USA; [2]Cold Spring Harbor Laboratory, Cold Spring Harbor, NY, USA; [3]Laboratory of Epigenetics and Chromatin Dynamics, Division of Gastroenterology and Hepatology, Department of Internal Medicine, Epigenomics Translational Program, Center for Individualized Medicine, Mayo Clinic, Rochester, MN, USA; [4]Department of Surgical Oncology, The University of Texas MD Anderson Cancer Center, Houston, TX, USA; [5]Department of Medicine, University of Illinois, Chicago, IL, USA; [6]Department of Surgery, Feinberg School of Medicine, Northwestern University, Chicago, IL, USA; [7]Jesse Brown VA Medical Center, Chicago, IL, USA; and [8]The Robert H. Lurie Comprehensive Cancer Center, Chicago, IL, USA.

[9] co-first authors

Supplemental Data

[*]**Address correspondence to**:
Krishan Kumar, Ph.D., or Hidayatullah G. Munshi, M.D.
Department of Medicine
Feinberg School of Medicine
Northwestern University
303 E. Superior Avenue
Lurie 3-220 (K. Kumar) or Lurie 3-117 (H.G. Munshi)
Chicago, IL 60611, USA.
Phone: 312.503.1151; E-mail: krishan.kumar@northwestern.edu (K. Kumar)
Phone: 312.503.2301; E-mail: h-munshi@northwestern.edu (H.G. Munshi)

SUPPLEMENTAL FIGURE LEGENDS

Supplemental Figure 1: BET inhibitors decrease IFN-γ-induced PD-L1 expression in PDAC cells PSCs. (**A, B**) AsPC1 cells and primary PSCs #2 were pre-treated with the BET inhibitors JQ1 (1 μM) or I-BET151 (1 μM) for 30 minutes and then treated with IFN-γ (0.2 μg/mL) for 24 hours. The effect on *PD-L1* mRNA expression was determined by qRT-PCR and the effect on PD-L1 protein expression was determined by FACS analysis. (**C**) Primary PSCs #1, the pancreatic stellate cell line, and CD18 and AsPC1 PDAC cells were pre-treated with the BET inhibitor JQ1 (1 μM) and then treated with IFN-γ (0.2 μg/mL) for 1 hour, 3 hours, or 6 hours. The effect on *PD-L1* mRNA expression was determined by qRT-PCR. The results are representative of three independent experiments. Bar graphs represent means +/- S.D. ***, $p<0.001$ relative to control samples.

Supplemental Figure 2: BRD4 knockdown decrease IFN-γ-induced PD-L1 expression in PSCs. Primary PSCs #2 were transfected with control siRNA or with siRNAs against BRD2, BRD3 or BRD4 for 48 hours. The cells were then treated with IFN-γ (0.2 μg/mL) for 24 hours. The effect on the individual *BRD* mRNAs and BRD proteins was determined by qRT-PCR and by Western blotting, and the effect on *PD-L1* mRNAs was determined by qRT-PCR. Bands of BRD and GAPDH proteins from three independent experiments were quantified by densitometry and data expressed as relative ratios. The gene expression results are representative of three independent experiments. Bar graphs represent means +/- S.D. ns, not significant; *, $p<0.05$; **, $p<0.01$; ***, $p<0.001$ relative to control samples.

Supplemental Figure 3: (**A**) **c-MYC knockdown does not decrease IFN-γ-induced *PD-L1* expression in PDAC cells.** CD18 and AsPC1 PDAC cells were transfected with control siRNA or with siRNA against c-MYC for 48 hours. The cells were then treated with IFN-γ (0.2 μg/mL) for

24 hours. The effect on *c-MYC* and *PD-L1* mRNA was determined by qRT-PCR. (**B**) **Effect of BET inhibitors on IFN-γ-induced *IRF1* expression in PDAC cells.** CD18 and AsPC1 cells were pre-treated with the BET inhibitors JQ1 (1 µM) or I-BET151 (1 µM) for 30 minutes and then treated with IFN-γ (0.2 µg/mL) for 4 hours. The effect on *IRF1* mRNA was determined by qRT-PCR. (**C**) **Effect of BET PROTAC on IFN-γ-induced IRF1 expression in stellate cells.** Primary PSCs and the pancreatic stellate cell line were pre-treated with the BET PROTAC ARV-825 (0.25 µM) for 30 minutes and treated with IFN-γ (0.2 µg/mL) for 4 hours. The effect on IRF1 was determined by qRT-PCR and by Western blotting. Bands of IRF1 and HSP90 proteins from three independent experiments were quantified by densitometry and data expressed as relative ratios. The gene expression results are representative of three independent experiments. Bar graphs represent means +/- S.D. ns, not significant; *, $p<0.05$; **, $p<0.01$ relative to control samples.

Supplemental Fig. 1

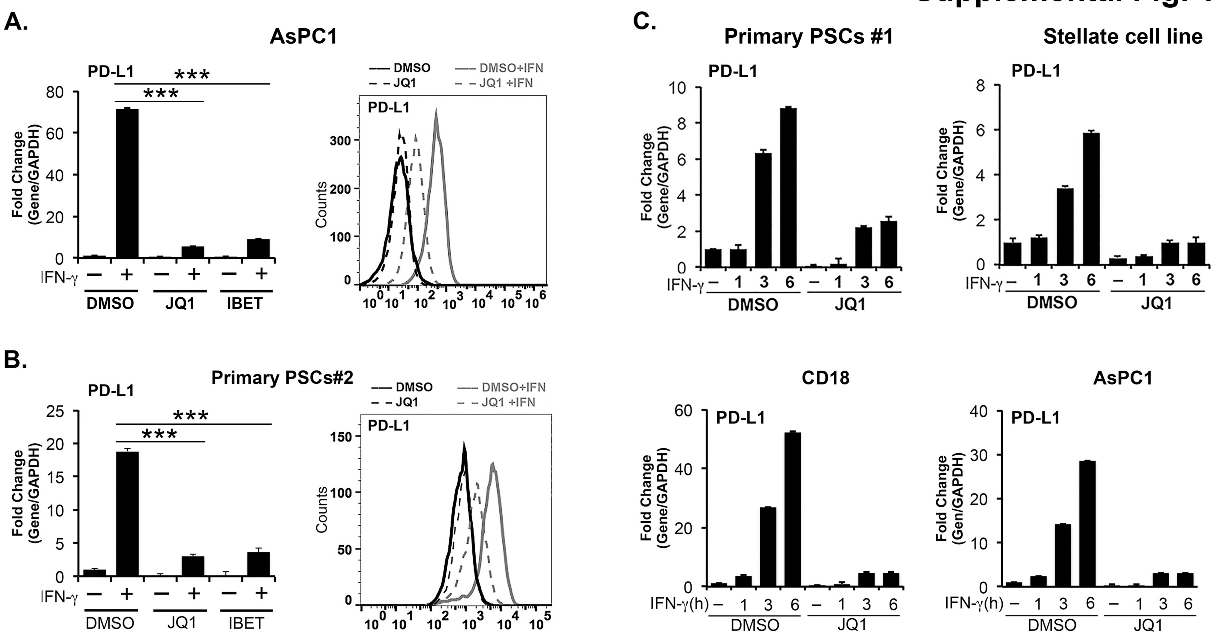

Supplemental Fig. 2

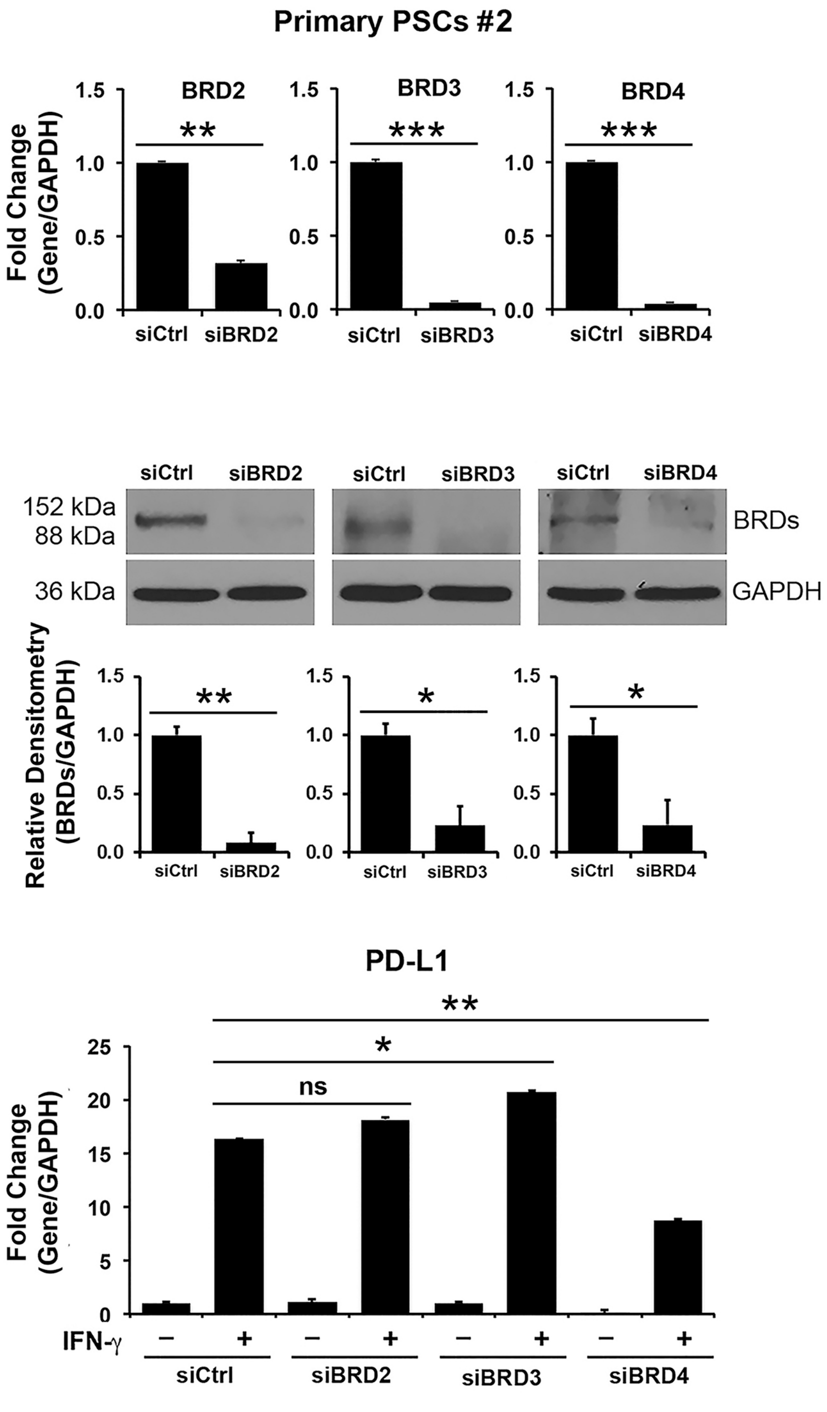

Supplemental Fig. 3

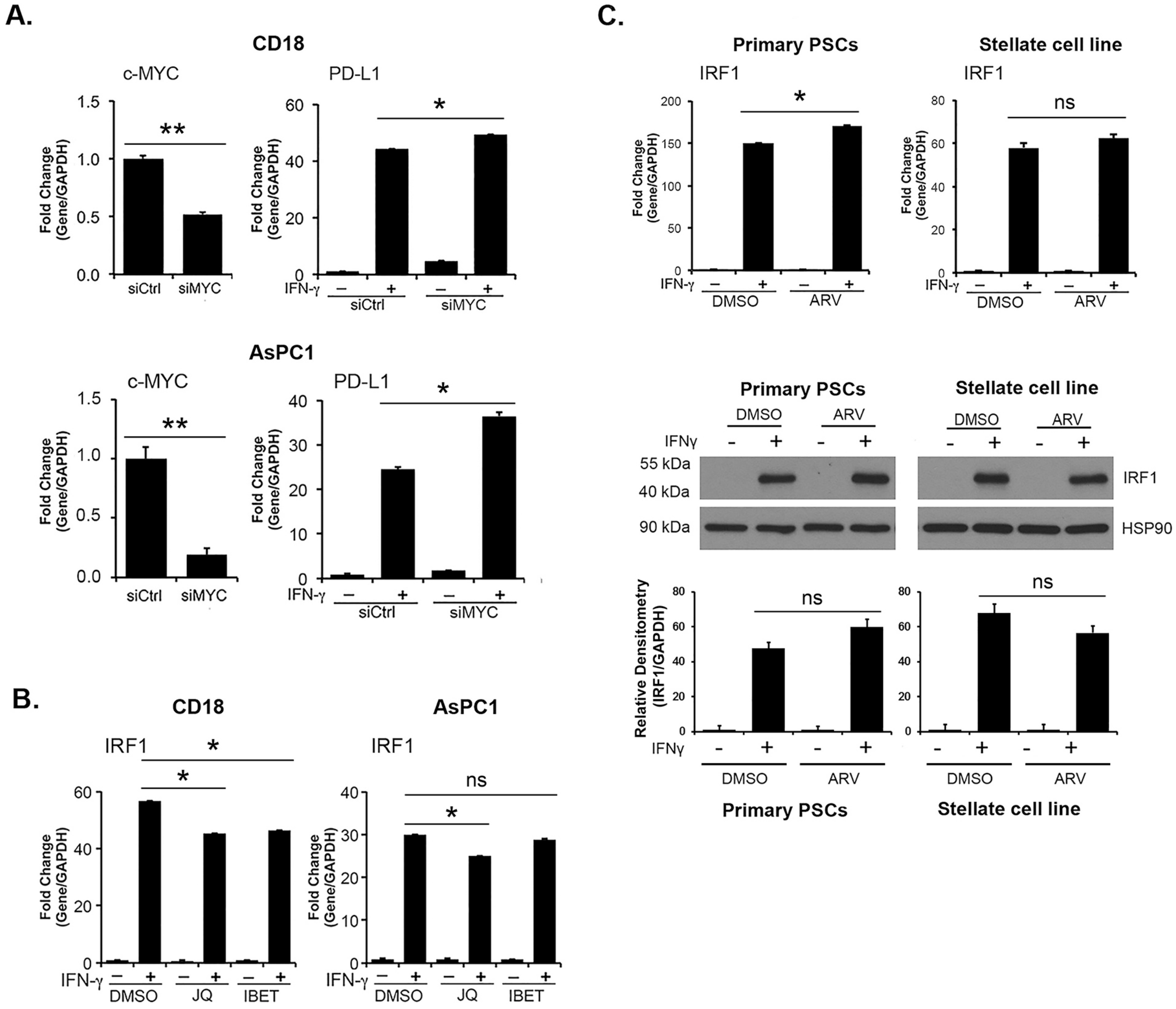

Supplement: Supplementary file 1 — Supplemental Figures 1-3 [file 41598_2018_31658_MOESM1_ESM.pdf]
